# Dynamic heterogeneity in complex interfaces of soft interface-dominated materials

**DOI:** 10.1038/s41598-019-39761-7

**Published:** 2019-02-27

**Authors:** Leonard M. C. Sagis, Bingxue Liu, Yuan Li, Jeffrey Essers, Jack Yang, Ahmad Moghimikheirabadi, Emma Hinderink, Claire Berton-Carabin, Karin Schroen

**Affiliations:** 1 Physics and Physical Chemistry of Foods, Wageningen University, Bornse Weilanden 9, 6708 WG Wageningen, The Netherlands; 2 ETH Zurich, Department of Materials, Polymer Physics, Leopold-Ruzicka-Weg 4, 8093 Zurich, Switzerland; 3 Beijing Advanced Innovation Center for Food Nutrition and Human Health, Key Laboratory of Functional Dairy, College of Food Science and Nutritional Engineering, China Agricultural University, 100083 Beijing, China; 4 Food Process Engineering Group, Wageningen University, Wageningen, 6700 AA The Netherlands

## Abstract

Complex interfaces stabilized by proteins, polymers or nanoparticles, have a much richer dynamics than those stabilized by simple surfactants. By subjecting fluid-fluid interfaces to step extension-compression deformations, we show that in general these complex interfaces have dynamic heterogeneity in their relaxation response that is well described by a Kohlrausch-Williams-Watts function, with stretch exponent β between 0.4–0.6 for extension, and 0.6–1.0 for compression. The difference in β between expansion and compression points to an asymmetry in the dynamics. Using atomic force microscopy and simulations we prove that the dynamic heterogeneity is intimately related to interfacial structural heterogeneity and show that the dominant mode for stretched exponential relaxation is momentum transfer between bulk and interface, a mechanism which has so far largely been ignored in experimental surface rheology. We describe how its rate constant can be determined using molecular dynamics simulations. These interfaces clearly behave like disordered viscoelastic solids and need to be described substantially different from the 2d homogeneous viscoelastic fluids typically formed by simple surfactants.

## Introduction

The behavior of complex interfaces, such as those found in living cells, is not well understood, unlike fluid-fluid interfaces stabilised with synthetic low molecular weight components, of which the dynamics are understood in great detail. Complex interfaces are known for their excellent physical stability, and nowadays found in many soft multiphase materials, such as emulsions, foams, aerosols, or microcapsules. The growing interest in bio-nanotechnology and biomimetic systems (e.g. artificial cells), has spurred substantial research on interface stabilization with nanoparticles (Pickering stabilization), protein (-complexes), or polymers. When stabilized by such materials, the macroscopic flow behavior of multiphase systems is markedly different from those stabilized by low molecular weight (LMW) surfactants. Their response to applied deformations can be highly nonlinear, and is often dominated by the properties of the interfaces. Multiphase systems that show this type of behavior are referred to as soft interface-dominated materials (SIDMs)^1^. The dynamics of SIDMs are affected by surface tension, in-plane mechanical properties (surface shear, surface dilatational, and surface Young’s modulus)^1,2^, and the resistance against out-of-plane deformations (bending and torsional viscosities and rigidities)^3,4^. These parameters affect the rise velocity of droplets in a fluid^5^, cause non-monotonic deformations of microcapsules in shear flows^6^, influence wave phenomena in stratified flows^7,8^, play a role in the suppression of the coffee ring effect^9^, are a factor in the dynamics of cells in arterial flows^10,11^, and many other multiphase systems^12^. A proper understanding of how these parameters affect macroscopic dynamics of SIDMs is often still lacking, and this has inspired a vast number of studies using a wide range of stabilizers, at both oil-water and air-water interfaces. Most of these studies do not use constitutive models to analyse mechanical data, and those that do, tend to use 2d generalizations of existing 3d phenomenological models (e.g., Maxwell, Burgers, or Jeffreys model)^1^, or variations of the Lucassen van den Tempel (LvdT) model^13,14^ (which link dilatational responses to adsorption-desorption processes). Such models assume the interface is a homogeneous 2d viscoelastic fluid, which in the non-deformed state is in equilibrium with its adjoining bulk phases, leading to adequate descriptions of the dynamics of interfaces stabilized by LMW surfactants. Here we show, using step dilatational experiments, imaging with atomic force microscopy (AFM), and computer simulations that the structure of interfaces in SIDMs is far more complex, and in general highly heterogeneous. The response of the interfaces investigated here to step expansions-compressions displays *dynamic heterogeneity*, a phenomenon commonly associated with disordered solids, and often related to spatial heterogeneity in the local relaxation kinetics^15,16^. We identify the dominant relaxation mode involved in this behavior and show how its rate constant can be determined using molecular dynamics simulations. Our results imply that the general behavior of these interfaces is fundamentally different from that of a 2d viscoelastic fluid.

## Results

### Step expansion and compression experiments

Dilatational properties of air-water and oil-water interfaces, stabilized by a wide range of ingredients (native, oxidized, and aggregated proteins, nanospheres, nanotubes; see Supplementary Materials for details), were determined using droplet tensiometers, in which a millimeter-sized droplet is formed at the tip of a motor syringe (see Methods). The shape of the droplet is captured with a camera, and image analysis is used to extract the surface stress, by solving the Young-Laplace equation. We use the term *surface stress* here, rather than surface tension, because in complex interfaces in-plane deviatoric stresses contribute to the total surface stress, when the interface is deformed^1^. The interface of the droplet was expanded/compressed by pumping fluid into/out of the droplet. First, stabilizers were allowed to adsorb at the interface (1000–6000 s). Then the area of the droplet was expanded by 10% (or 20%), and the change in surface stress was monitored for 1000–3000 s. Subsequently the area was compressed by 10% (or 20%), and again the change in surface stress was monitored. In our tests, none of the interfaces showed buckling or delamination during compression. In all cases the Young-Laplace equation gave a good fit to the droplet profile. For all stabilizers the change in surface stress *γ*(*t*), as a function of time was a smooth function (i.e. no avalanche-type relaxation), and was best described by an expression which combines a Kohlrausch-Williams-Watts stretched exponential term with a regular exponential:

1 $$\gamma (t)=a{e}^{-{(t/{\tau }_{1})}^{\beta }}+b{e}^{-t/{\tau }_{2}}+c$$

Here τ_1_ is the relaxation time associated with stretched exponential behavior, β is the stretch exponent, and *a*, *b*, and *c* are constants. The regular exponential in equation (1) describes an aging process of the interface, which is also present in the signal when the interface is not compressed or expanded. Complex stabilizers, such as proteins or nanoparticles, are known to have adsorption behavior with extremely long time tails, which are typically associated with rearrangements of the interfacial structure, occurring after adsorption; τ_2_ is a characteristic time for these rearrangements. Introduction of this term decouples the actual relaxation behavior from aging processes of the interface. Its presence indicates that all interfaces studied here are essentially *non-equilibrium* systems.

Table 1 shows values for β and τ_1_, for all samples tested here, and some published in recent literature^17–21^. For the latter, only compression data are available (for *a, b, c*, and τ_2_ see Supplementary Material). We observe that in extension all stabilizers have a value for β in the range of 0.4–0.6, and that the value in compression is higher (0.5–1.0), which implies there is an *asymmetry* in the dynamics between compression and extension. A value of β<1 is a signature of what is commonly referred to as *dynamic heterogeneity*^15,16^, a well-established phenomenon in 3d disordered solids, and in those systems related to spatial heterogeneity of local relaxation kinetics. It has rarely been observed for fluid-fluid interfaces (see Table 1), and only been identified as such for synthetic polymers, spread at air-water interfaces, above the glass temperature of the polymer^17,18^. The asymmetry in the relaxation response we observe here, has to our knowledge not been previously reported. Similar asymmetries have however been observed in large amplitude oscillatory dilatational deformations of complex interfaces^22^.

**Table 1:** Stretch exponent β and relaxation time τ_1_ for various stabilizers at air-water (a/w) or oil-water (o/w) interfaces; C (mg/ml) denotes the concentration of the stabilizer in the bulk phase.

Stabilizer	C (g/l)	Type of interface	Expansion		Compression		Ref.
			*β*	τ_1_(s)	*β*	τ_1_(s)	
Nanospheres (NS)	0.5	o/w	0.55	19.5	1	62.9	—
Big nanospheres (BNS)	0.5	o/w	0.66	10.7	1	34.1	—
Nanotubes	0.5	o/w	0.68	17.2	0.85	29.0	—
Cross-linked nanotubes	0.5	o/w	0.54	12.1	1	68.0	—
Cross-linked NS	0.5	o/w	0.63	19.2	1	12.0	—
Cross-linked BNS	0.5	o/w	0.58	14.8	1	17.0	—
WPI (native) 10% Δ	20.0	a/w	0.56 ± 0.04	19.6 ± 1.1	0.69 ± 0.01	20.0 ± 2.2	—
WPI (native) 20% Δ	20.0	a/w	0.54 ± 0.04	13.7 ± 1.2	0.60 ± 0.01	17.2 ± 0.6	—
WPI aggregates 10% Δ	20.0	a/w	0.52 ± 0.02	20.3 ± 4.3	0.57 ± 0.00	23.8 ± 1.3	—
WPI aggregates 20% Δ	20.0	a/w	0.55 ± 0.03	16.5 ± 1.7	0.59 ± 0.02	17.3 ± 1.9	—
PPI (native)	0.5	a/w	0.42 ± 0.06	17.9 ± 9.1	0.73 ± 0.07	18.0 ± 6.2	—
PPI (24 hour ox.)	0.5	a/w	0.59 ± 0.10	19.1 ± 4.3	0.56 ± 0.21	30.9 ± 19.8	—
P4HS	a)	a/w	—	—	~0.5	~100	17
PMMA	a)	a/w	—	—	0.5–0.6	10^3^–10^4^	18
β-lactoglobulin pH 8.3	a)	a/w	—	—	~0.6	500–2000	19
β-lactoglobulin pH 6.0	a)	a/w			~0.6	500–1000	19
β-casein pH 8.3	a)	a/w	—	—	0.5–0.7	~1000	19
Polyglycerol ester (PGE)	0.1	a/w	—	—	0.6 ± 0.1	243 ± 67	20
E-coli lipids + FtsZ-ZipA	a)	a/w	—	—	<1	~100	21

a) Stabilizer was spread on the interface in a Langmuir trough; a range of surface coverages or surface pressures were tested.

The fact that we observe dynamic heterogeneity for such a wide range of stabilizers, with different sizes and structures, at both air-water and oil-water interfaces, suggests that this behavior is not limited to interfaces with spread polymers, but is in fact a generic behavior for complex fluid-fluid interfaces.

**Figure 1:**
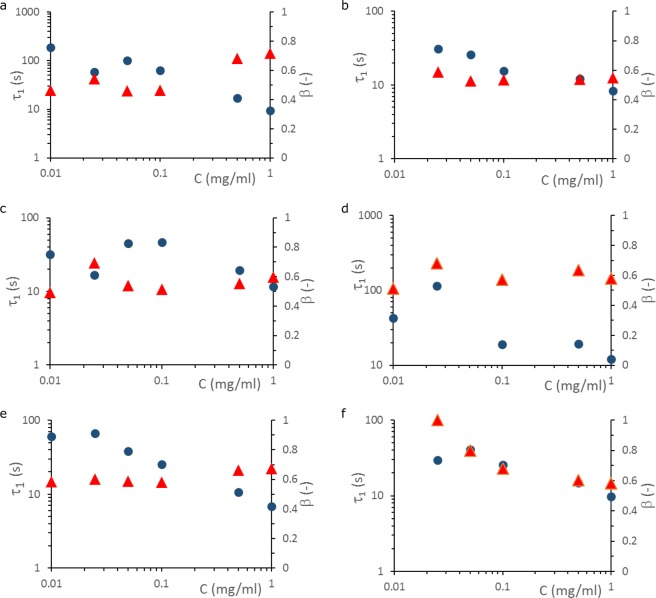
Relaxation time τ_1_ (spheres) and stretch exponent β (triangles) as a function of concentration of stabilizer in the bulk. Plots are for o/w interfaces stabilized by (**a**) nanotubes, (**b**) cross-linked nanotubes, (**c**) small nanospheres, (**d**) cross-linked small nanospheres, (**e**) big nanospheres, and (**f**) cross-linked big nanospheres.

For P4HS and PMMA spread at the air-water interface, in compression, Hilles *et al*.^17^ and Meastro *et al*.^18^ observed regular exponential behavior (β = 1) at the lowest surface concentrations (dilute regime), suggesting the interfacial structure was homogeneous^17^. For higher surface concentrations β started to decrease, to a value close to 0.5, which suggests they were observing the onset of dynamic heterogeneity. For oil-water interfaces stabilized by nanotubes, small nanospheres, big nanospheres, and their cross-linked versions (which have a higher stiffness), β in expansion was nearly independent of bulk concentration, and τ_1_ decreased with increasing concentration (Fig. 1). The surface pressures $(\pi ={\gamma }_{0}-\gamma (t)$, where *γ*_0_ is the surface tension of the bare fluid-fluid interface) for our nanoparticle stabilized interfaces were all in the concentrated regime (see Fig. S2), and hence we do not see a strong dependence of β on surface pressure, except for the cross-linked big nanospheres, where we see a similar onset as observed by Hilles *et al*.^17^ and Meastro *et al*.^18^.

Note that the values for τ_1_ in compression, obtained from the literature^17–21^, all are much longer than the ones we measured. We attribute this to the fact that in these studies the stretch exponential term was fitted to the entire relaxation curve (i.e. no regular exponential term was included to correct for the aging of the interface). Let us now examine the origin of the observed stretched exponential behavior.

### Emergence of dynamic heterogeneity

Klafter and Shlesinger^16^ presented a number of scenarios which can lead to dynamic heterogeneity. They show that this behavior emerges when relaxation occurs through a large number of parallel processes, with a wide distribution of relaxation times. Alternatively, it emerges when relaxation occurs through a hierarchy, or cascade of processes, where one level has to relax, before the next sublevel can.

To see how these scenarios can lead to stretched exponential behavior in fluid-fluid interfaces, we must first take a closer look at the mechanisms through which the surface stress can relax. There are three basic mechanism through which relaxation can occur: (1) in-plane momentum transfer, (2) mass transfer of stabilizer between interface and bulk phase, and (3) momentum transfer between bulk and interface. In the Supplementary Material we discuss in detail how these mechanisms can be described quantitatively, within the Gibbs dividing surface framework. Here we merely provide a qualitative description. In-plane momentum transfer involves the lateral rearrangement of the interfacial microstructure. The second mechanism is described by the well-known LvdT model^13,14^, and is the main mode involved in dilatational behavior of interfaces stabilized by simple soluble surfactants. In expansion the dilation of the surface can drive mass transfer of stabilizer from the bulk to the interface. In compression, the reverse process may occur. The third mechanism has received little attention in the literature, but is nevertheless a very important one, since it controls how the interfacial dynamics affect the bulk dynamics. In continuum mechanics it is described by the boundary condition that couples the bulk and interfacial momentum balances (see Supplementary Material), and its rate constant is a tensorial transfer coefficient referred to as the friction tensor. The nanoparticles, the native and aggregated WPI, and oxidized PPI tested here all have a β between 0.55 and 0.6 (Table 1), which is very close to the stretch exponent found for relaxation phenomena in 3d disordered solids^15^. This may be an indication that the behavior of the interface is much closer to a 3d subsystem than a 2d one, implying that all three mechanisms could be involved in the relaxation process, and responsible for the observed dynamic heterogeneity.

The interactions between stabilizers adsorbed at an interface are significantly different, both in nature, strength and range, from those in the bulk phase^1,23–26^. So even when stabilizers form stable solutions/dispersions in the bulk, they may form segregated interfacial structures after adsorption, in which dense clusters coexist with less dense regions. Such a disordered solid structure is markedly different from the homogeneous liquid-like structure typically assumed for simple surfactant stabilized interfaces. All three primary relaxation mechanisms would be affected by the degree of structural heterogeneity of the interface: a wide cluster size distribution would result in a distribution of relaxation times for in-plane rearrangement and momentum transfer between bulk and interface. The rate of adsorption of stabilizer to the interface is affected by its local surface density, and the characteristic time associated with adsorption/desorption would also need to be replaced by a distribution of times. In terms of the scenarios presented by Krafter and Schlesinger^16^, it is most likely that the observed dynamic heterogeneity emerges through parallel processes, and is a result of structural heterogeneity.

### Interfacial microstructure

**Figure 2:**
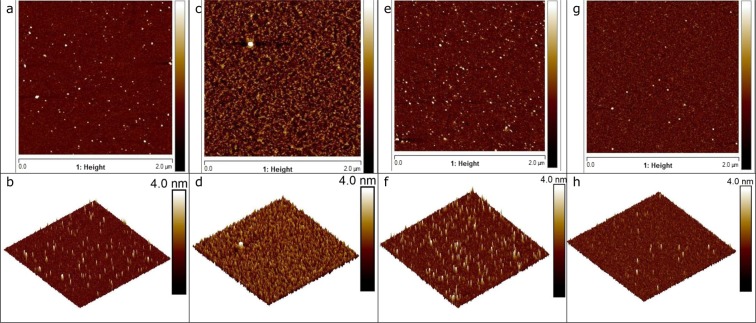
Images of native WPI, native PPI, and 24 h oxidized PPI spread at the air-water interface, produced with AFM after LB deposition on mica sheets; (**a**,**b**) 2d and 3d view of native WPI, at surface pressure π=15 mN/m; (**c**,**d**) 2d and 3d view of WPI at π=25 mN/m; (**e**,**f**) 2d and 3d view of native PPI at π=20 mN/m; (**g**,**h**) 2d and 3d view of 24 h oxidized PPI at π=15 mN/m; surface area of the AFM images is 2 × 2 μm; Color code for the upper row of images is identical to the lower row.

To confirm whether structural heterogeneity is indeed at the basis of the observed dynamic heterogeneity, we performed imaging of the interfaces of some of our systems with AFM, after deposition of the interface on a mica substrate at specific surface pressures (see Methods). In Fig. 2 we have plotted 2d and 3d AFM images of native WPI, native PPI, and 24 hour oxidized PPI, spread at the air water interface. In all three systems we see substantial spatial heterogeneities in the interfacial structure, and the structure is markedly different from that of a homogeneous surfactant film. Particularly for WPI at a low surface pressure of 15 mN/m (Fig. 2a,b) the interfacial film is clearly segregated and densely clustered regions can be observed which alternate with regions where the film is much thinner. At a higher surface pressure of 25 mN/m (Fig. 2c,d) the surface is still clearly heterogeneous, but clusters are now more densely packed. The structure of the WPI interfaces, at a surface pressure of 25 mN/m (Fig. 1c) show a striking resemblance with the structure of three- dimensional heat-set WPI gels, produced at the same pH, which are known to have a fractal nature^27^. The surfaces with native PPI and 24 hour oxidized PPI also show significant heterogeneity in the interfacial structure (Fig. 2e,h). We may therefore conclude that structural heterogeneity is at the basis of the observed dynamic heterogeneity. This would also explain why in compression we see that all β are higher than in extension, and for the nanoparticles the stretched exponential behavior even completely disappears. When mass exchange between bulk and interface is sufficiently slow, compression leads to a significantly denser and more homogeneous structure, and this is accompanied by a loss of dynamic heterogeneity in the relaxation behavior.

There are several other indicators that these types of interfaces are heterogeneous, and behave as soft disordered viscoelastic solids. Their surface shear and dilatational moduli are often (nearly) independent of frequency, in the tested frequency range (0.001–10 Hz)^28–30^. The loss tangents for these interfaces tend to be low (≤0.1), and in large amplitude dilatational studies they often show asymmetries in Lissajous-Bowditch plots, displaying softening in extension, and hardening in compression^22,30^. In surface shear they may display yielding behavior at a critical surface stress^31^. All these are indicators of soft disordered solid behavior.

**Figure 3:**
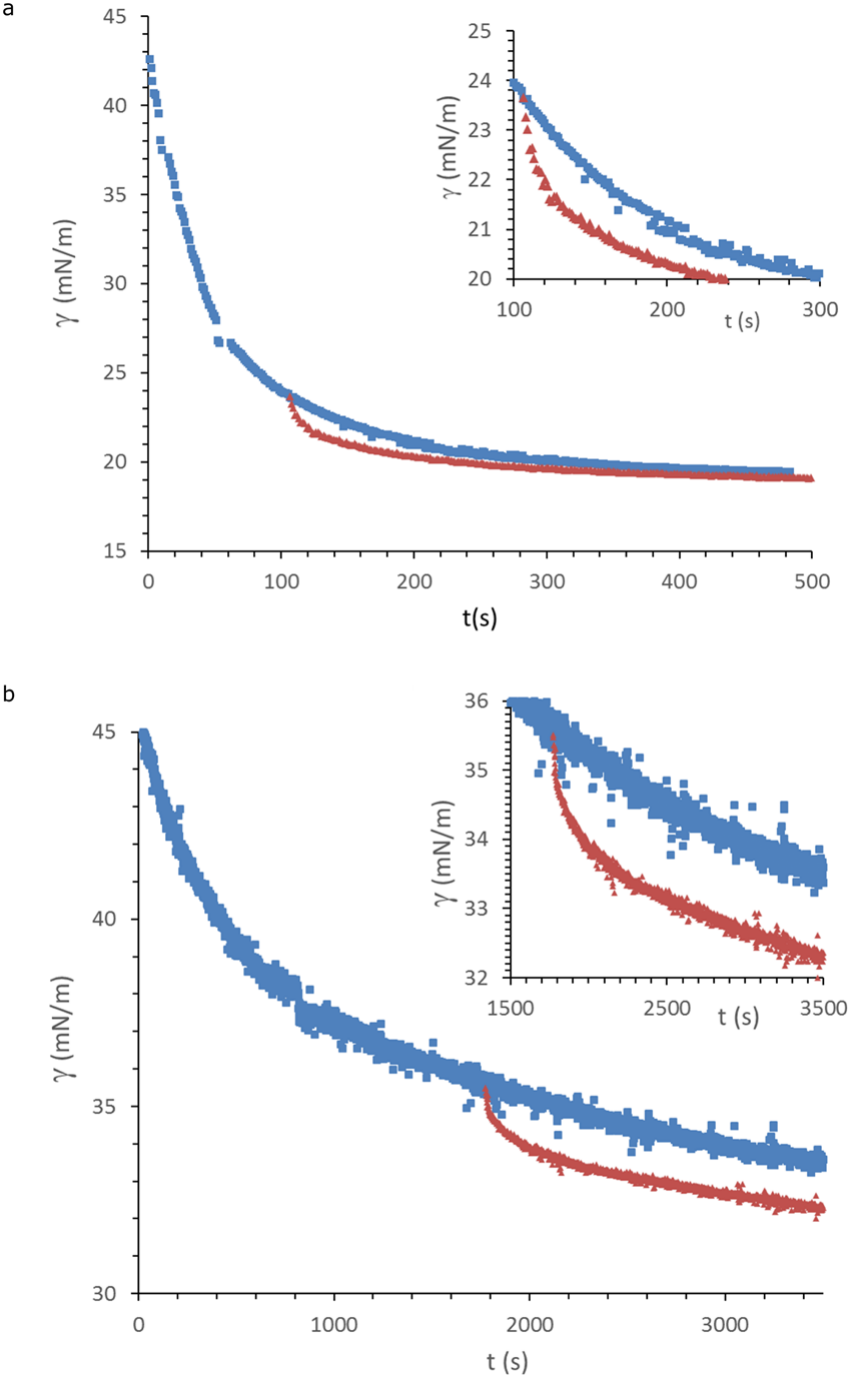
γ(t) during the adsorption phase (blue squares), and the (shifted) relaxation phase after 10% step expansion (red triangles), for a) nanospheres (c = 0.05 g/l), and b) nanotubes (c = 0.025 g/l); inserts are a blow-up of the expansion phase.

To determine which relaxation mode is dominant, we have plotted the surface stress as a function of time for the initial adsorption phase in Fig. 3 (for nanospheres and nanotubes), and the surface stress after step expansion, shifted in time, so that the maximum surface stress after the step coincides with the same surface stress in the adsorption phase. We immediately see that the relaxation we observe is much faster than the adsorption process (τ_1_~10–20 s), indicating that mass transfer is not a dominant factor here. Of course, this comparison is valid only, when the deformations are affine. If only the weaker dilute regions would be deformed, patches of (nearly) bare interface could be created. In Fig. 3 we see that adsorption to a bare interface is however much faster than the relaxation. Moreover, for native and aggregated WPI the magnitude of deformation does not significantly affect the response. These observations indicate the deformation is indeed close to affine. Of all systems tested here, the surface properties of interfaces stabilized by native and aggregated WPI tend to be the most sensitive to the magnitude of deformation (see also Supplementary Materials). We therefore assumed affine deformations for all tested systems.

Since mass transfer between bulk and interface is not a dominant factor here, this leaves only in-plane momentum transfer or momentum transfer between bulk and interface as possible relaxation mechanisms. All relaxation times we determined in expansion were around 10–20 s. If the dominant mode here was in-plane momentum transfer, we would expect to see features in the frequency dependence of the dilatational modulus in the range around 0.01 Hz. As noted above, for most of the complex stabilizers tested here, the moduli are nearly constant in that frequency range. Based on this, we believe the dominant relaxation mode here is momentum transfer between the interface and the bulk phase, a mode which is typically completely ignored in experimental dilatational studies. This would also explain why for this wide range of stabilizers (native, oxidized, and aggregated proteins, nanospheres, nanotubes) which differ significantly in structure, we observe very similar stretch exponents and τ_1_ relaxation times. For these solid-like films, momentum transfer between the interfacial film and the adjoining bulk phases will primarily be mediated through the outer contact regions of the film and will be relatively less dependent on the details of its interior structure, and hence less dependent on the architecture of the stabilizer.

In the next section we show how the rate constant for this mode can be determined using molecular dynamics simulations.

### Simulations

**Figure 4:**
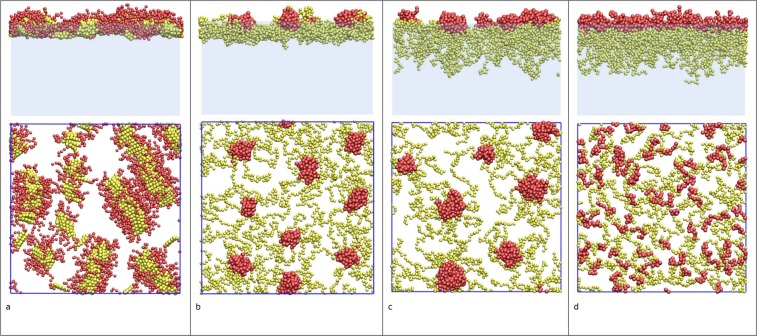
Structures formed by block copolymers at the liquid-vapor interface: (**a**) linear strands (effective dimension *d*_*e*_=1), (**b**) in-plane clusters (1≤*d*_*e*_≤2), (**c**) 3d clusters (2≤*d*_*e*_≤3), and (**d**) 3d films (*d*_*e*_=3). The top row shows the side view and the bottom row shows the top view of the interfacial film.

To gain a better understanding of the origin of the structural inhomogeneities, and the resulting dynamic heterogeneity, we performed Molecular Dynamics (MD) simulations on one of the types of stabilizers listed in Table 1: model block-copolymers adsorbed at liquid-vapor interfaces. In these MD simulations we varied the surface concentration, the architecture of, and the interactions between the block-copolymers (see Methods). These variations created a wide range of microstructures, with effective dimensions between 1 and 3, such as linear strands (Fig. 4a), in-plane clusters (Fig. 4b), 3d clusters (Fig. 4c), and 3d films (Fig. 4d) (more details provided in the Supplementary Materials). This shows that even relatively simple stabilizers, can produce a wide range of heterogeneous surface phases. Similar simulations for large numbers of native globular protein molecules are not yet available because of computational limitations^32^. Recent simulations on interfaces stabilized by ellipsoidal colloidal particles, interacting through capillary interactions^33^, do however show similar types of heterogeneous structures, varying from randomly aggregated clusters at low surface coverage, to strand-like structures at high surface coverage.

Experimentally, such strand-like interfacial structures have been observed for ellipsoidal particles^25^ and polymers spread at air-water interfaces^34^. Polymers^34^ and spherical colloidal particles at air-water interfaces^24^ are also known to form various types of clusters.

**Figure 5:**
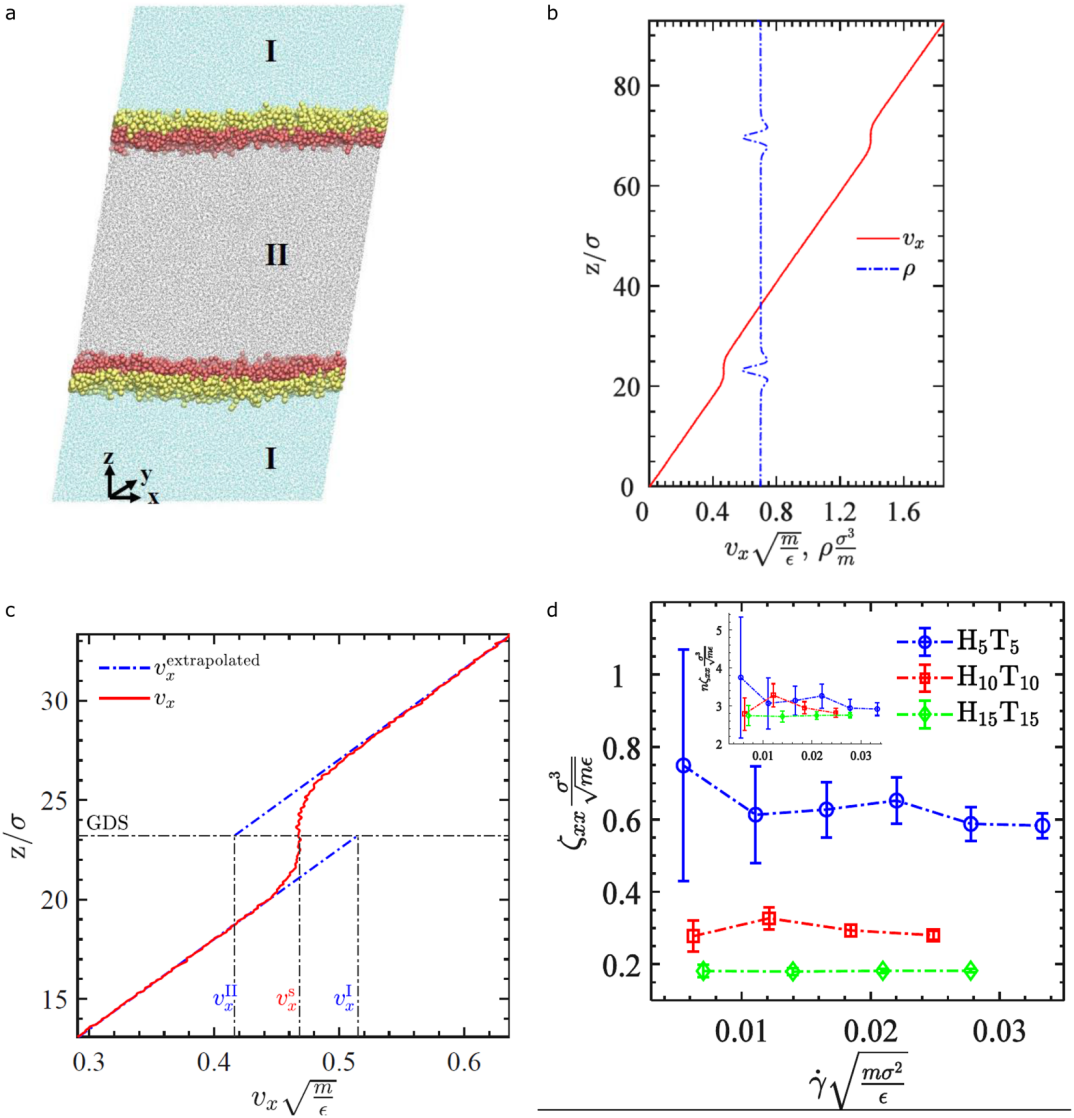
Nonequilibrium molecular dynamics simulations of the structure and momentum transfer coefficient, for an interface stabilized by H_n_T_n_ block-copolymers (n = 5, 10, 15), subjected to a shear field with the gradient direction perpendicular to the interface: (**a**) geometry of the problem, (**b**) density and x-component of velocity as a function of vertical position z; (**c**) extrapolated bulk velocities and surface velocity; (**d**) momentum transfer coefficient ξ_xx_ as a function of shear rate; the inset shows the product of ξ_xx_ with block size n.

For the block-copolymer system we also used Nonequilibrium Molecular Dynamics (NEMD) simulations to arrive at an estimate of the rate constant for momentum exchange between interface and the bulk phase (see Methods). We subjected a thin film of phase II (Fig. 5a), between two fluids I, with interfaces stabilized by block-copolymers H_n_T_n_ (n = 5, 10, 15), to shear with the gradient direction perpendicular to the interface (the z-direction). We solved for the shear stress *σ*_*xz*_ (see Supplementary Materials) and velocity profile (Fig. 5b), and extracted the extrapolated bulk velocity, *v*_*x*_, and surface velocity ${v}_{x}^{s}$ (Fig. 5c). For the symmetric case simulated here (I and II have equal bulk viscosities and densities), the shear stress and difference between extrapolated and surface velocity are related by (see Supplementary Materials)

2 $${\sigma }_{xz}={\zeta }_{xx}\cdot ({v}_{x}-{v}_{x}^{s})$$

here ζ_*xx*_ is the effective transfer coefficient for exchange of momentum between bulk and interface. Equation (2) allows us to calculate this coefficient from the simulation results (Fig. 5d). The coefficient decreases for increasing block size n. At the densities we have chosen, the velocity in the interfacial layer is nearly constant in the z-direction, and as a result the difference between extrapolated bulk velocity and surface velocity scales nearly linear with the thickness of the interfacial layer (and hence block size). As a result, the product of ζ_*xx*_ with block size n is almost constant (see inset Fig. 5d) for the three simulated polymers. As we pointed out in the previous section, for any stabilizer where the interface is solid-like, momentum transfer is primarily mediated through the thin contact regions of the film with the bulk, and hence relatively insensitive to the detailed architecture of the stabilizer.

Our simulations show how the momentum transfer mechanism can be quantified, and can act as a basis for the design of experiments to measure this coefficient, using methods from the field of nano-tribology^35,36^.

## Discussion

Our results show conclusively, that interfaces with a complex microstructure, such as protein, polymer, or particle-stabilized interfaces in general display significant dynamic heterogeneity and asymmetry between compression and expansion, which cannot appropriately be described by currently used 2d viscoelastic fluid models, or the LvdT model^13,14^. Their behaviour is more similar to that of a viscoelastic disordered solid, and requires alternative constitutive models to describe their behavior. In some cases, particularly when the response of the interface is still in the linear response regime, the dynamic heterogeneity can still be captured satisfactorily using multimode^37^ or fractional derivative Maxwell models^38^. But for larger deformations, where nonlinearities and yield stress phenomena start playing a role, more complex models are needed. Much can be learned here from the community working on microcapsules with shells consisting of cross-linked polymers, which tend to have *elastic* solid behaviour. An example here is the recent work by Hegemann *et al*.^39^, in which the pendant drop method, which we used here as well, was extended to droplets with nonlinear Hookean elastic behaviour. Alternative models such as Mooney-Rivlin were also tested.

Our results also show that for all interfaces tested here, the dominant mode for the stretched exponential relaxation is momentum transfer between interface and bulk, a mode that has so far been largely ignored in the literature on surface dilatational rheology. The friction factors that quantify this transfer can be determined from NEMD simulations, and could be measured using nano-tribology methods^35,36^.

Although mass transfer between bulk and interface is slow for the stabilizers we investigated here, its effects on relaxation cannot always be ignored, and for some systems all modes of relaxation need to be accounted for simultaneously. Such models can be created either in the 2d Gibbs dividing surface, or 3d diffuse interface framework^12^. In the former this can be done by starting from a detailed microscopic model that is subsequently coarse-grained to a 2d model^40^, describing in-plane dynamics, and transfer processes between bulk and interface. Both approaches require a fundamental change in how complex interfaces are currently being viewed. The deeper insight in interfacial structure and dynamics thus obtained should be taken as a starting point for the design of any multiphase system with complex interfaces.

## Methods

### Nanoparticle preparation

*Nanotubes*. Firstly, α-Lactalbumin (30 g/L) was dissolved in 75 mM pH 7.5 Tris-HCl buffer. Then Bacillus licheniformis protease (BLP) (BLP to α-lactalbumin weight ratio, 1:14) and CaCl_2_ (Ca^2+^ to α-lactalbumin molar ratio, 2:1) were added to the α-Lactalbumin solution. The mixed solution was passed through a 0.22 μm filter. The reaction mixture was heated at 50 °C for 1 h allowing the self-assembly of partially hydrolyzed α-Lactalbumin into nanotubes. The gelation of the reaction mixture after 1 h indicated the formation of a nanotube network.

*Nanospheres*. α-Lactalbumin (1 g/l) and BLP (BLP to α-lactalbumin weight ratio, 1:25) were dissolved in 75 mM pH 7.5 Tris-HCl buffer. The reaction mixture was passed through a 0.22 μm filter and heated at 50 °C for 30 min allowing the self-assembly of hydrolyzed α-Lactalbumin into nanospheres. Relatively small nanospheres (diameter ~20 nm) were formed for low protein concentrations of 1 g/l. Relatively larger nanospheres (diameter ~200 nm) were formed for high protein concentration of 30 mg/ml for equal BLP to protein ratio (see TEM images in Supplementary Materials).

*Cross-linked nanoparticles*. 1 g/l nanoparticles (nanotubes or nanospheres) were mixed with 2.5% glutaraldehyde (protein to glutaraldehyde molar ratio, 1:50). The glutaraldehyde was added gradually in three equal amounts with a time interval of 10 minutes, allowing for a slow crosslinking reaction at 25 °C. During the cross-linking reaction, a vertical mixing apparatus was used, and the rotational speed was 30 rpm. The cross-linking results in a stiffening of the particles.

### Protein sample preparation

*Pea protein isolate (PPI) solutions*. A stock protein solution was prepared by mixing 6 grams of PPI, purity 85% (NUTRALYS S85F, Roquette, France), with 94 grams of phosphate buffer solution (10 mM, pH 7.0) and stirring it at 250 rpm overnight at 4 °C. The obtained protein solution was subsequently centrifuged for 30 minutes at 16,000 g and 20 °C. The supernatant was collected and subjected to the same centrifugation step a second time, after which the soluble protein fraction (the supernatant) was stored at −25 °C until further analysis.

*Whey protein isolate (WPI) solutions and WPI aggregate dispersions*. Whey protein isolate (WPI) purity 98% (Davisco Foods international, France) was used as received. WPI solutions were prepared by dissolving 2.5% (w/w) WPI in a sodium phosphate buffer (20 mM, pH 7). The solution was centrifuged at 22,000 g for 30 min. The supernatant was filtered using a 33 mm ø hydrophilic PES syringe filter with 0.45 µm pore size (Millipore, Billerica, MA, USA). The dry matter content was determined by evaporation at 105 °C in the oven. The solution was diluted with sodium phosphate buffer to a 2% (w/w) WPI solution. Preparation of WPI aggregates was carried out according to van Leusden *et al*.^41^. A 10% (w/w) WPI in MQ solution was stirred overnight. The solution was adjusted to pH 8.0 using 1 M NaOH. Subsequently, the solution was heated at 80 °C for 30 min while stirring. Afterwards, the solution was cooled with tap water and diluted with sodium phosphate buffer (25 mM, pH 7.0) to a 2% (w/w) solution. The solutions were adjusted to pH 7.0 with 1 M HCl or 1 M NaOH and stored at 4 °C.

### Surface dilatational measurements

*Step relaxation for PPI samples*. For air-water interfaces stabilized by PPI samples a Tracker (Teclis, Longessaigne, France) automated drop tensiometer was used to study their response. PPI solutions were loaded into a 250 μL syringe, which was connected to an 18-gauge Teflon coated needle. Solutions were diluted with sodium phosphate buffer (10 mM, pH 7.0) to a protein concentration of 0.5 mg/ml prior to loading. Each measurement started with 5 hours of equilibration of the surface stress at a droplet area of 15 mm^2^. Subsequently, the droplet area was increased to 18 mm^2^ (20% expansion, applied in 2 s), where it was kept at for 2 hours. After that the droplet area was decreased to 14.4 mm^2^ (20% compression, applied in 2 s), where it stayed for 2 hours. During expansion and compression of the droplet, surface stress and droplet area were continuously monitored. The area was kept constant using a DIP-controlled feedback system based on the parameters registered by the camera and image analysis software. Fitting of equation (1) to the surface stress results was done with MATLAB R2016b, utilizing the curve fitting tool. Fitting was performed on data from the first 1000 s, starting from the maximum (minimum) value of the surface stress in extension (compression). Reported parameters are an average of two batches, with three measurements per batch.

*Step relaxation for WPI*. WPI-stabilized interfaces were subjected to step deformations in a Sinterface PAT-1M profile analysis tensiometer (Sinterface Technologies, Berlin, Germany) at 20 °C. A pending drop was formed at the tip of a needle (ø 1.96 mm) in a glass curvet. The surface area of the drop was equilibrated at 20, 22 or 24 mm^2^ for 180 min to obtain quasi-equilibrium conditions prior to the step. Steps of 10% (20 to 22–22 to 19.8 mm^2^) and 20% (20 to 24–24 to 19.2 mm^2^) expansion or compression were performed (step time 2 s) and the surface stress was monitored for one hour. Measurements were done at least in triplicate. The surface tension of the first 1000 seconds after the step change were fitted to equation (1) using the Curve Fitting App in Matlab v16b for Windows 7 OS (Mathworks, Natrick, MA, USA).

*Step relaxation for nanoparticles*. Step relaxation was performed with a Data Physics OCA-20 (Germany). Before the measurement, nanoparticles solutions were diluted to the required concentration (0.01, 0.025, 0.05, 0.1, 0.5, 1 g/l) and dialysed against deionized water. The initial surface area of the water/hexadecane interface was 25 mm^2^. The interfaces were allowed to reach equilibrium for about 5000 s for low NP concentrations (0.01, 0.025 g/l), and about 2000 s for high NP concentrations (0.5, 1.0 g/l). Then we expanded the interfacial area by 10% in 2 seconds. Relaxation of the surface stress was monitored for 2500 s at low NP concentrations and 1000 s at high NP concentrations. Then we compressed the surface area by 10%, again in 2 seconds, and monitored the surface stress. The temperature was kept at 25 °C during all experiments.

### Langmuir-Blodgett films

Films were prepared using a 273 mm^2^ Langmuir film balance (Langmuir-Blodgett Trough KN 2002, KSV NIMA/Biolin Scientific Oy, Espoo, Finland) at room temperature. The sub-phase was sodium phosphate buffer (PPI: 10 mM, pH 7.0; WPI: 20 mM, pH 7). Protein layers were formed by spreading 200 µl of 0.02% (w/w) buffered protein solution on the surface. The monolayers were equilibrated for 30 minutes before compression. The surface pressure of the monolayer was measured using a Wilhelmy plate (39.44 mm perimeter). The monolayer was compressed by Teflon barriers moving at 5 mm/min. The films were transferred on freshly cleaved mica substrate (Highest Grade V1 Mica, Ted Pella, Redding, CA, USA) at constant surface pressure and 1 mm/min speed. The films were dried in a desiccator and stored at room temperature. The films were produced in duplicate.

### Determination of the interfacial structure

The interfacial structure was determined using an atomic force microscope (AFM, MultiMode 8-HR, Bruker, Billerica, MA, USA). AFM images of the Langmuir-Blodgett films were recorded in tapping mode using Scanasyst-air model non-conductive pyramidal silicon nitride probes (Bruker, Billerica, MA, USA) with a normal spring constant of 0.40 N/m. A lateral scan frequency of 0.977 Hz was employed for all topographical images. The lateral resolution was set to 512 × 512 pixels in a scan area of 2 × 2 µm. At least two locations were observed for each sample to ensure a good representativeness. The AFM images were analysed using Nanoscope Analysis 1.5 software (Bruker, Billerica, MA, USA).

### Molecular dynamics simulations

In this section, we present details about both equilibrium and nonequilibrium MD simulations performed to determine interfacial microstructures and the friction coefficient of the interfacial layer.

#### Equilibrium molecular dynamics (MD) simulations

Our model systems are comprised of a monoatomic Lennard-Jones liquid, W (water-like particles), and nonionic linear diblock- or triblock-copolymers, H_30_T_10_ and T_5_H_10_T_5_, where H segments (hydrophilic) have more affinity towards the solvent than T segments (hydrophobic). A freely-jointed bead-spring model with a harmonic bond potential *u*_*b*_(equation 3) for block-copolymers, and a truncated and shifted Lennard-Jones potential *u*_*ij*_(equation 4) for all non-bonded pairs within the system are used. All species (W particles and block-copolymer segments H and T) are considered to be of the same size *σ* and mass *m*. As well as the above-mentioned bond and non-bonded potentials, we have introduced a harmonic angle *u*_*a*_(equation 5) and dihedral potentials *u*_*d*_ (equation 6) to the middle block of triblock-copolymers, to make them rigid, so they could form linear strands after adsorbing to the interface. All non-bonded and bonded interaction potential parameters are given in Table S2 and S3 in the Supplementary Materials.

3 $${u}_{b}=\frac{1}{2}{k}_{b}{(l-{l}_{0})}^{2}$$

4 $${u}_{ij}=\{\begin{array}{ll}4{\varepsilon }_{ij}({(\frac{\sigma }{{r}_{ij}})}^{12}-{(\frac{\sigma }{{r}_{ij}})}^{6})-4{\varepsilon }_{ij}({(\frac{\sigma }{{r}_{{\rm{cut}},ij}})}^{12}-{(\frac{\sigma }{{r}_{{\rm{cut}},ij}})}^{6}), & {r}_{ij}\le {r}_{{\rm{cut}},ij}\\ 0, & {r}_{ij} > {r}_{{\rm{cut}},ij}\end{array}\}$$

5 $${u}_{a}=\frac{1}{2}{k}_{a}{({\theta }_{ijk}-{\theta }_{0})}^{2}$$

6 $${u}_{d}={k}_{d}(1+d\,\cos \,{{\varphi }}_{ijkl})$$

We have used LAMMPS^42^ to perform all MD simulations in the canonical ensemble. Each simulation consists of three main steps followed by a sampling interval. In the first step, we consider a state point $T=0.723\,\varepsilon /{k}_{B},\rho \approx 0.8{\sigma }^{-3}$ that has been studied before^43^, for a pure liquid consisting of W particles in a simulation box with dimensions ${L}_{x}={L}_{y}=50.56\sigma$ and ${L}_{z}=46.77\sigma$. An integration time step of $0.006\,\sigma \sqrt{m/\varepsilon }$ is used to perform the simulation for 10^5^ time steps. After reaching equilibrium, we increase the simulation box length in the z-direction to *L*_*z*_ = 92.96*σ* (so that we have a liquid slab with a thickness of 46.77 in the middle of the simulation box) and continue the simulation for an additional 2·10^5^ time steps, until having two bare liquid-vapor interfaces normal to the z-direction. We place 150 diblock-copolymers H_30_T_10_ (or 400 triblock-copolymers T_5_H_10_T_5_) randomly in the interfacial region by selecting 40 (or 20) adjacent W particles from either top or the bottom interface for each block-copolymer molecule, connecting them together and changing their identity to H and T segments, such that the overall number density in the simulation box does not change. Afterwards, we let the simulation run for another 3·10^5^ time steps and check thermodynamic properties such as pressure tensor components to ensure equilibrium is reached. In the sampling interval, we continue the simulation for 2.5·10^5^ time steps and collect samples once every 50 steps for data analysis. Additional details on the simulation results are given in the Supplementary Materials.

#### Nonequilibrium molecular dynamics (NEMD) simulations

We have considered symmetric surfactants at liquid-liquid interfaces by introducing an oil-like liquid phase (namely O particles) to the system which interacts through a truncated and shifted LJ potential (equation 4) with all other species (W, H, T). All particles in the system have the same mass *m* and size *σ*. The energy depth ε_*ij*_ for every pair in the system is the same and equal to ε. Identical pairs as well as H-W and T-O have the same ${r}_{{\rm{cut}},ij}=2.5\sigma$ (and hence have both repulsive and attractive branches of the LJ potential) while for all other pairs ${r}_{{\rm{cut}},ij}={2}^{\frac{1}{6}}\sigma$ (and hence are purely repulsive). Symmetric diblock-copolymers H_n_T_n_ (where n=5, 10, 15) are considered to be freely-jointed with a FENE bond potential between consecutive beads

7 $${u}^{{\rm{FENE}}}(l)=\frac{-{k}_{{\rm{FENE}}}}{2}{{l}_{0}}^{2}\,\mathrm{ln}(1-{(\frac{l}{{l}_{0}})}^{2})$$

where ${k}_{{\rm{FENE}}}=30\frac{\varepsilon }{{\sigma }^{2}}$ and ${l}_{0}=1.5\sigma$. The same system size $({L}_{x}={L}_{y}=50.56\sigma ,\,{L}_{z}=92.96\sigma )$ and state point $(T=1.0\,\varepsilon /{k}_{B},\rho \approx 0.7{\sigma }^{-3})$ which we have studied in reference^44^ are considered. LAMMPS^42^ was used to perform NEMD simulations in the canonical ensemble. Each simulation consists of three main steps followed by a sampling interval. In the first step, we perform equilibrium MD simulation for a system consisting of a layer of O particles sandwiched between two layers of W particles such that the total number of O and W particles are the same. We let the system relax for 10^5^ steps until having two O-W interfaces normal to the z-direction in equilibrium. For each block copolymer chain H_n_T_n_, we select randomly 2n adjacent O and W particles from the interfacial region (where the first n particles are of the W type and the rest are of the O type) and connect them together and change their identity to H and T segments. We run the simulation for another 1.5·10^5^ steps to reach equilibrium. At this point, we start the NEMD simulation by shearing the simulation box (with the desired shear rate) in the direction parallel to the interface (x-direction) for 5·10^5^ steps to ensure reaching a steady state. Samples are then collected once every 50 time steps for the final 2.5·10^5^ sampling interval. More details and simulation results are given in the Supplementary Materials.

## Supplementary information

Dynamic heterogeneity in complex interfaces of soft interface dominated materials

## Data Availability

The datasets generated during and/or analyzed during the current study are available from the corresponding author on request.
