# Supplementary material for: Dynamic heterogeneity in complex interfaces of soft interface-dominated materials
Source: Sci Rep. 2019 Feb 27;9:2938. doi: 10.1038/s41598-019-39761-7 (PMC6393553; doi:10.1038/s41598-019-39761-7)
Supplement: Supplementary file 1 — Dynamic heterogeneity in complex interfaces of soft interface dominated materials [file 41598_2019_39761_MOESM1_ESM.pdf]

Supplementary Information to:

Dynamic heterogeneity in complex interfaces of soft interface dominated materials

Leonard M. C. Sagis[1,2,†*], Bingxue Liu[3†], Yuan Li[3,*], Jeffrey Essers[1,4], Jack Yang[1], Ahmad Moghimikheirabadi[2], Emma Hinderink[4], Claire Berton-Carabin[4], Karin Schroen[4]

[1] Physics and Physical Chemistry of Foods, Wageningen University, Bornse Weilanden 9, 6708 WG Wageningen, The Netherlands.

[2] ETH Zurich, Department of Materials, Polymer Physics, Leopold-Ruzicka-Weg 4, 8093 Zurich, Switzerland

[3] Beijing Advanced Innovation Center for Food Nutrition and Human Health, Key Laboratory of Functional Dairy, College of Food Science and Nutritional Engineering, China Agricultural University, 100083, Beijing, China.

[4]Food Process Engineering Group, Wageningen University, Wageningen 6700 AA, The Netherlands

† These authors contributed equally to this work

* Corresponding authors

Leonard.sagis@wur.nl, Tel: +31(0)317485023

yuanli@cau.edu.cn, Tel: +86(0)1062738589

Relaxation mechanisms in multiphase systems with complex interfaces

In the main text of this paper we have outlined three basic mechanisms by which relaxation of the surface stress after a step deformation of an interface can occur: 1) in-plane rearrangement of the interfacial microstructure, 2) mass transfer of stabilizer between the interface and the bulk phases, and 3) momentum transfer between the interface and the adjoining bulk phases. In this section we will discuss how these mechanisms can be described in the context of continuum mechanics.

In continuum mechanics there are two basic frameworks to describe the dynamics of interfaces in multiphase systems. These are the diffuse interface model, and the Gibbs dividing surface model[1-3]. In the former, interfaces are treated as three-dimensional regions of finite thickness, in which densities and material properties vary rapidly but continuously, from their value in one bulk phase to their value in the adjoining bulk phase. In the Gibbs dividing surface model the interface is modelled as a two-dimensional surface, placed sensibly within the interfacial region[1-3]. Bulk densities and material properties are extrapolated into the interfacial region up to the dividing surface, and the difference between the actual fields and extrapolated ones is accounted for by assigning excess densities and excess surface properties to the interface (e.g. excess densities like the surface mass density or surface momentum density, and excess properties, such as a surface viscosity, a surface thermal conductivity, or surface diffusion coefficient). Here we will focus only on the description of interfacial dynamics in the Gibbs dividing surface framework.

For an N-component multiphase system, the system variables in the bulk phases are the total mass density, ρ , the momentum density,$\boldsymbol{m} = \rho\boldsymbol{v}$ (where $\boldsymbol{v}$ is the bulk velocity field), the internal energy density $\bar{U}$ (J/m^3), and the component densities $\rho_{(J)} = \rho\omega_{(J)}$ (J=1,…,N-1;$\omega_{(J)}$ is the mass fraction of component J). By assigning a surface excess to each of these fields, associated with the dividing surfaces, an additional set of system variables is introduced, which are the surface mass density, ρ^s (kg/m^2), the surface momentum density,$\boldsymbol{m}^s = \rho^s\boldsymbol{v}^s$ (where $\boldsymbol{v}^s$ is the surface velocity), the surface internal energy density $\bar{U}^s$ (J/m^2), and the surface mass densities of the individual components, $\rho^s_{(J)} = \rho^s\omega^s_{(J)}$ (J=1,…,N-1;$\omega^s_{(J)}$ is the surface mass fraction of component J). Including these surface excess fields in the formulation of the conservation laws (conservation of mass, conservation of momentum, and conservation of energy), gives us, apart from the familiar balances for the bulk phases (the differential mass balance, the differential momentum balance, and the differential energy balance), a set of differential balance equations describing the time evolution of the surface excess variables[1-3]. These balances are often referred to as jump balances[1-3] and are coupled to the balances for the bulk variables, through a set of boundary conditions. Here we will briefly illustrate this for the component mass balance and momentum balance.

In a system without chemical reactions, the principle of conservation of mass for each component in the system implies that the time rate of change of the total mass of that component is zero. Mathematically, we can describe this as[1-3]:

$$\frac{d}{dt}\left[\int_R \rho\omega_{(J)} dV + \int_\Sigma \rho^s\omega^s_{(J)} d\Omega\right] = 0, \tag{S.1}$$

where the first integral represents the total mass of component J associated with the bulk phase (with domain R), and the second integral equals the total mass of J associated with the dividing surfaces (with domain Σ). The symbols dV and $d\Omega$ denote a volume and area integration, respectively. Evaluating the time derivative gives us for each bulk phase in the system[1-3]

$$\rho \frac{d_b \omega_{(J)}}{dt} = -\nabla \cdot \boldsymbol{j}_{(J)}, \tag{S.2}$$

Here $\boldsymbol{j}_{(J)} = \rho_{(J)}(\boldsymbol{v}_{(J)} - \boldsymbol{v})$ is the mass flux vector for component J, and ∇ is the three-dimensional gradient operator. For the surface mass fraction of J associated with the dividing surface we find:

$$\rho^s \frac{d_s \omega^s_{(J)}}{dt} = -\nabla_s \cdot \boldsymbol{j}^s_{(J)} - [\![\rho(\omega_{(J)} - \omega^s_{(J)})(\boldsymbol{v} - \boldsymbol{v}^s) \cdot \boldsymbol{n} + \boldsymbol{j}_{(J)} \cdot \boldsymbol{n}]\!] \tag{S.3}$$

The vector $\boldsymbol{j}^s_{(J)} = \rho^s_{(J)}(\boldsymbol{v}^s_{(J)} - \boldsymbol{v}^s)$ is the surface mass flux vector for component J, the operator ∇_s is the surface gradient operator [1-3]. The material derivatives in (S.2) and (S.3) are defined as[1-3]

$$\frac{d_b \psi}{dt} = \frac{\partial \psi}{\partial t} + (\nabla \psi) \cdot \boldsymbol{v} \qquad \frac{d_s \psi^s}{dt} = \frac{\partial \psi^s}{\partial t} + (\nabla_s \psi^s) \cdot \dot{\boldsymbol{y}}. \tag{S.4}$$

Here $\dot{\boldsymbol{y}}$ is the intrinsic surface velocity, defined as $\dot{\boldsymbol{y}} \equiv \boldsymbol{v}^s - \boldsymbol{u}$, where $\boldsymbol{u}$ is the speed of displacement of the interface. The double brackets in the jump mass balance are defined as[1-3]

$$[\![\psi \boldsymbol{n}]\!] = \psi^{\mathrm{I}} \mathbf{n}^{\mathrm{I}} + \psi^{\mathrm{II}} \boldsymbol{n}^{\mathrm{II}}. \tag{S.5}$$

Here ψ^{I} and ψ^{II} are, respectively, the value of ψ in bulk phase I and II, evaluated at the dividing surface. The vector $\boldsymbol{n}^I$ is the unit vector normal to the dividing surface, pointing in the direction of phase I (and hence, $\mathbf{n}^{\mathrm{I}} = -\boldsymbol{n}^{\mathrm{II}}$). Equation (S.3) describes, for every point on the dividing surface, the time rate of change of the surface mass fraction of component J, as a result of (in order of appearance on the right-hand side of the equation) in-plane surface diffusion, convective transport between bulk and interface, and diffusive transfer between bulk and interface. Equation (S.2) and (S.3) are coupled by two boundary conditions (one for bulk phase I, one for phase II), and for an isothermal system these conditions are given by (M=I, II)[4]

$$\boldsymbol{j}^M_{(J)} \cdot \boldsymbol{n}^M + \rho^M_{(J)}(\boldsymbol{v}^M - \boldsymbol{v}^s) \cdot \boldsymbol{n}^M = -\Lambda^M_{(J)}(\tilde{\mu}^M_{(J)} - \tilde{\mu}^s_{(J)}), \tag{S.6}$$

Where $\Lambda^M_{(J)}$ is the coefficient for mass transfer of component J between bulk phase M and the dividing surface, $\tilde{\mu}^M_{(J)}$ is the chemical potential of J in phase M, evaluated at the interface; $\tilde{\mu}^s_{(J)}$ is the surface chemical potential of J. When convective transfer between interface and bulk can be neglected, (S.6) reduces to

$$\boldsymbol{j}^M_{(J)} \cdot \boldsymbol{n}^M = -\Lambda^M_{(J)}(\tilde{\mu}^M_{(J)} - \tilde{\mu}^s_{(J)}), \tag{S.7}$$

and we see this is in essence a constitutive equation for the mass flux between bulk and interface. This last equation is needed to describe stress relaxation processes by the second mechanism, i.e. exchange of mass between interface and bulk phase. When the system is at equilibrium, for each component J, $\tilde{\mu}^I_{(J)} = \tilde{\mu}^{II}_{(J)} = \tilde{\mu}^s_{(J)}$, and there is no diffusive exchange between interface and bulk. When the interface is expanded, we dilute the concentration of stabilizer J at the interface, and hence lower its surface chemical potential. This will drive a diffusive flux from the bulk phase to the interface. When we compress the interface, we increase the surface potential of J, and this drives a mass flux from the interface to the bulk.

In a similar manner, we find that the conservation of momentum implies for each of the bulk phases that (in the absence of external force fields)[1-3]

$$\rho \frac{d_b \boldsymbol{v}}{dt} = \nabla \cdot \boldsymbol{T}, \tag{S.8}$$

and for the dividing surfaces

$$\rho^s \frac{d_s \boldsymbol{v}^s}{dt} = \nabla_s \cdot \boldsymbol{T}^s - [\![\rho(\boldsymbol{v} - \boldsymbol{v}^s)(\boldsymbol{v} - \boldsymbol{v}^s) \cdot \boldsymbol{n} - \boldsymbol{T} \cdot \boldsymbol{n}]\!] \tag{S.9}$$

Here $\boldsymbol{T}$ is the stress tensor in the bulk phase, and $\boldsymbol{T}^s$ is the surface stress tensor. These two tensors are in general split in a hydrostatic and deviatoric contribution:

$$\boldsymbol{T} = -p\boldsymbol{I} + \boldsymbol{\sigma}, \qquad \mathbf{T}^s = \gamma \boldsymbol{P} + \boldsymbol{\sigma}^s. \tag{S.10}$$

Here p is the pressure, $\mathbf{I}$ is the three-dimensional unit tensor, $\boldsymbol{\sigma}$ is the extra stress tensor in the bulk, γ is the surface tension, $\mathbf{P}$ is the two-dimensional unit tensor, and $\boldsymbol{\sigma}^s$ is the surface extra stress tensor. Substituting (S.10) in (S.9) we find[1-3]

$$\rho^s \frac{d_s \boldsymbol{v}^s}{dt} = \nabla_s \gamma + 2\gamma H \boldsymbol{n} + \nabla_s \cdot \boldsymbol{\sigma}^s - [\![\rho(\boldsymbol{v} - \boldsymbol{v}^s)(\boldsymbol{v} - \boldsymbol{v}^s) \cdot \boldsymbol{n} + P\boldsymbol{n} - \boldsymbol{\sigma} \cdot \boldsymbol{n}]\!]. \tag{S.11}$$

Here H denotes the local mean curvature of the interface. This last expression, often referred to as the jump or interface momentum balance, is essentially a generalization of the Young-Laplace equation for the static configuration of a curved interface. It describes for every point on the dividing surface the time rate of change of surface momentum as a result of (in order of appearance on the right-hand side of the equation) surface tension gradients (Marangoni stresses), curvature induced stresses, in-plane deviatoric stresses, and, in the double bracket term, the inertial, hydrostatic, and deviatoric stresses exerted by the adjoining bulk phases on the interface. The in-plane deviatoric stress term can be used to model stress relaxation by the first mechanism, in-plane microstructural rearrangements. By choosing an appropriate 2d viscoelastic model, such as a (multi-mode) Maxwell, Jeffreys, or Burgers model, or alternatively, a structural model which links surface stresses directly to changes in the interfacial microstructure, one can quantify this contribution to the surface relaxation dynamics. Our data presented in the main text suggest that for the systems we tested, the in-plane rearrangement and mass transfer mechanisms are unlikely to be responsible for the observed dynamics. The only remaining mechanism is momentum transfer between the interface and the adjoining bulk phases, and this can be modelled through the two boundary conditions that couple the bulk differential momentum balance (S.8) to the differential jump momentum balance (S.11). These boundary conditions are given by (M=I, II)[4]

$$\boldsymbol{\sigma}^M \cdot \boldsymbol{n}^M - \rho^M \boldsymbol{v}^M (\boldsymbol{v}^M - \boldsymbol{v}^s) \cdot \boldsymbol{n}^M = \sum_{N=\mathrm{I}}^{\mathrm{II}} \boldsymbol{\zeta}^{M,N}\, T^s \cdot \left(\frac{\boldsymbol{v}^N}{T^N} - \frac{\boldsymbol{v}^s}{T^s}\right), \tag{S.12}$$

where the tensors $\boldsymbol{\zeta}^{M,N}$ are friction tensors[4]. The specific form of this boundary condition is suggested by the entropy balance. Equation (S.12) describes momentum transfer across interfaces as a two-step process (see Scheme S.1), in which momentum in phase I is first transferred to the interface (with coefficient $\boldsymbol{\zeta}^{I,I}$), and then transferred further to phase II (with coefficient $\boldsymbol{\zeta}^{I,II}$). Conversely, momentum in phase II is first transferred to the interface (with coefficient $\boldsymbol{\zeta}^{II,II}$), and then transferred onward to phase I (with coefficient $\boldsymbol{\zeta}^{II,I}$).

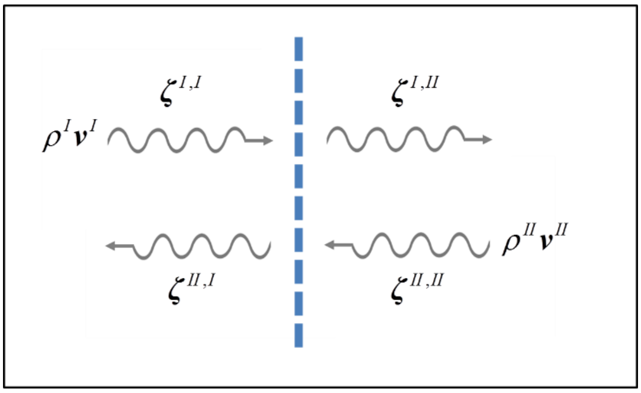

Scheme S.1. Explanation of the friction tensors appearing in equation (S.12).

For an isothermal system in which inertial stresses can be neglected, Equation (S.12) reduces to

$$\boldsymbol{\sigma}^M \cdot \boldsymbol{n}^M = \sum_{N=\mathrm{I}}^{\mathrm{II}} \boldsymbol{\zeta}^{M,N} \cdot (\boldsymbol{v}^N - \boldsymbol{v}^s), \tag{S.13}$$

and we see that the structure of these expressions is very similar to that in Equation (S.7): we have a flux for exchange (in this case momentum) between the bulk and the interface, which is equal to a coefficient (the friction tensor) times a jump in momentum (for the mass flux the equivalent jump was a jump in chemical potential). Equation (S.13) can be used to determine an estimate of the friction coefficients through NEMD simulations. Let us consider a flat interface in the xy-plane, between two Newtonian fluids I, and II. The direction perpendicular to the interface is denoted by z. In this configuration the location of the dividing surface is given by z=0, and the unit normal vector is given by $(0,0,1)$. If we now impose a shear deformation on this system, which causes a flow field of the form $(v_x^M(z),0,0)$ and $(v_x^s,0,0)$, then (S.13) reduces to (at z=0):

$$\sigma_{xz}^I = \zeta_{xx}^{I,I} \cdot (v_x^I - v_x^S) + \zeta_{xx}^{I,II} \cdot (v_x^{II} - v_x^S), \tag{S.14}$$

$$\sigma_{xz}^{II} = \zeta_{xx}^{II,I} \cdot (v_x^I - v_x^S) + \zeta_{xx}^{II,II} \cdot (v_x^{II} - v_x^S), \tag{S.15}$$

For the symmetric case (same fluids for phase I and II, and a symmetric surfactant) we can simplify this by using $\zeta_{xx}^{I,I} = \zeta_{xx}^{II,II}$, $\zeta_{xx}^{I,II} = \zeta_{xx}^{II,I}$, and $(v_x^I - v_x^s) = -(v_x^{II} - v_x^s)$, to find

$$\sigma_{xz}^I = \zeta_{xx} \cdot (v_x^I - v_x^S), \qquad \zeta_{xx} = \zeta_{xx}^{I,I} - \zeta_{xx}^{I,II} \tag{S.16}$$

Here ζ_{xx} is the effective friction coefficient of the layer. By simulating this system for an out-of-plane shear deformation, we can determine the velocity profile $v_x^M(z)$ and v_x^s along with the shear stress σ_{xz} . By extrapolating $v_x^M(z)$ to z=0 and substituting this extrapolated value and the value for v_x^s in (S.16) we obtain an estimate of the effective friction coefficient of the layer.

Characteristics of the nanotubes and nanospheres

In Figure S1 we present TEM images of the nanotubes and nanospheres. These were produced with a JEM-1400, JEOL. First, the samples were diluted to 1g/l, then 10 μl of sample was placed on a carbon film supported by a copper grid. After 1 min 30 s, filter paper was used to remove most of the fluid from the edge of the copper mesh. Then a drop of 10 μL water was added to wash away impurities on the copper mesh for 1min. Finally, 10 μL 3% uranyl acetate was used to negatively stain the samples and removed after 90 s.

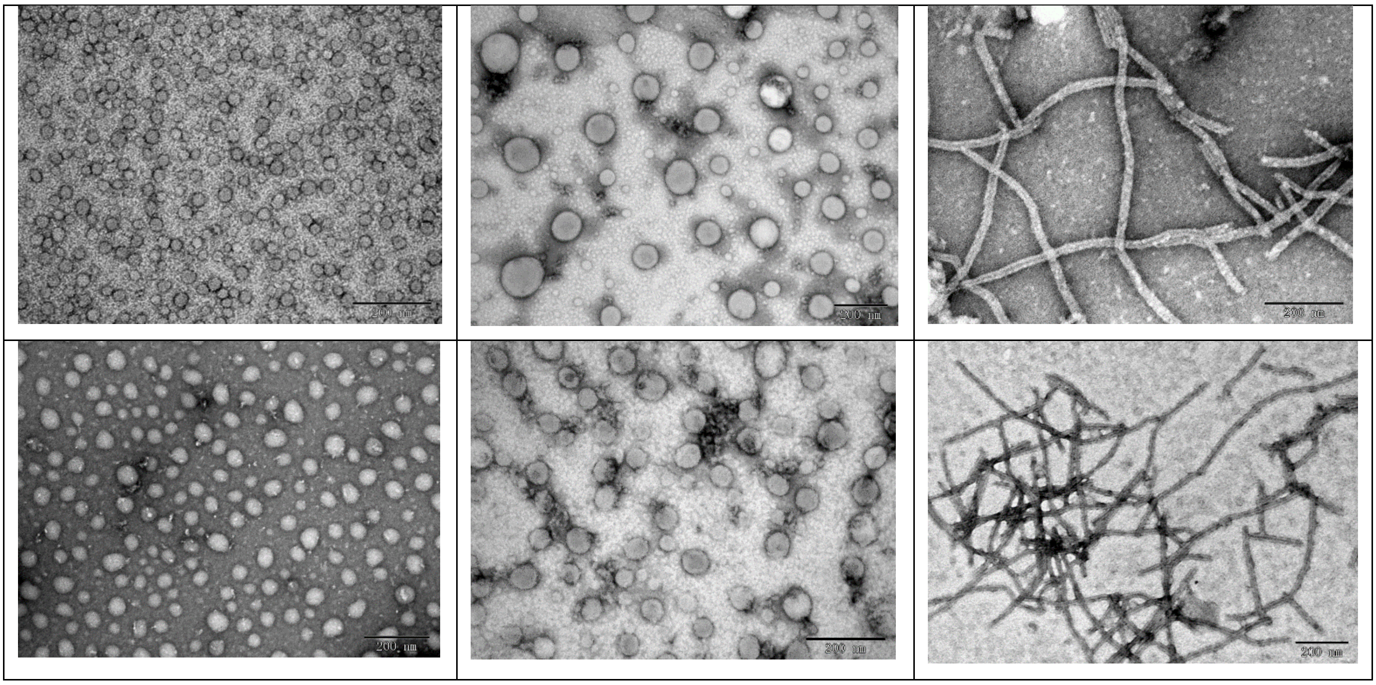

Figure S1. Top row (left to right): TEM images of nanospheres, big nanospheres, and nanotubes; bottom row (left to right): cross-linked nanospheres, cross-linked big nanospheres, and cross-linked nanotubes. Scale bars represent 200 nm.

Step expansion – compression experiments

Figure S2 illustrates the step expansion – compression experiments, for a step change of 20%, for a PPI stabilized a/w interface. Delamination or buckling effects are not observed in the compression phase, and the profile of the droplet satisfies the Young-Laplace equation.

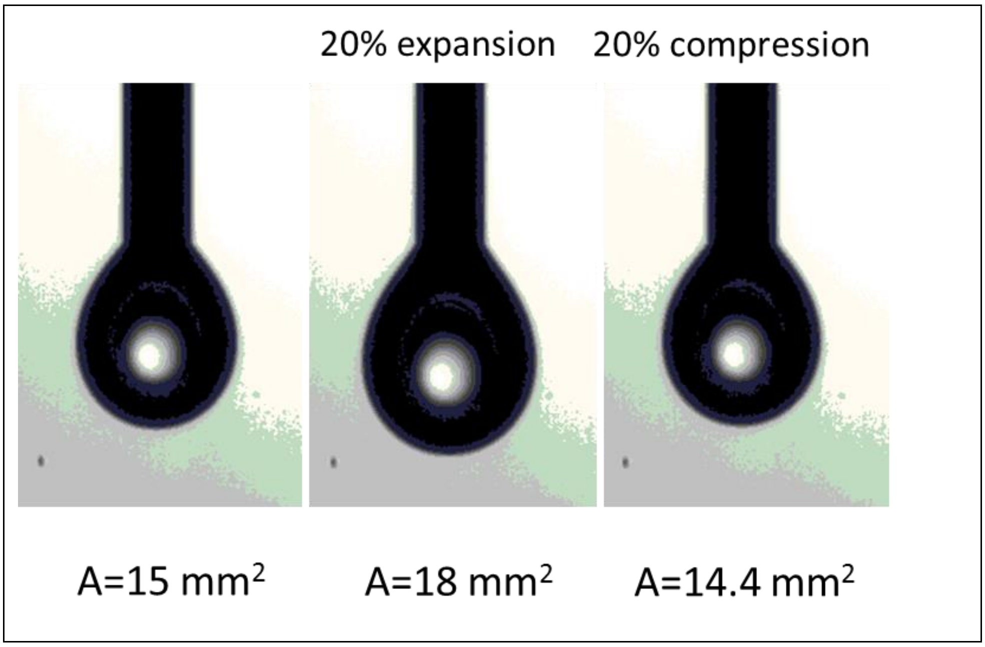

Figure S2. Droplet profiles in 20% expansion and 20% compression for a PPI stabilized a/w interface.

Strain dependence of dilatational moduli

To give an indication of the dependence of the surface properties on the magnitude of deformation, Figure S3 shows the dilatational storage and loss modulus for WPI and PPI-stabilized a/w interfaces, determined in an oscillatory strain sweep. The moduli of PPI-stabilized interfaces are nearly independent of strain amplitude. The storage modulus of WPI-stabilized interfaces shows a mild strain dependence. This drop in modulus is apparently too small to significantly affect the relaxation dynamics after a step deformation.

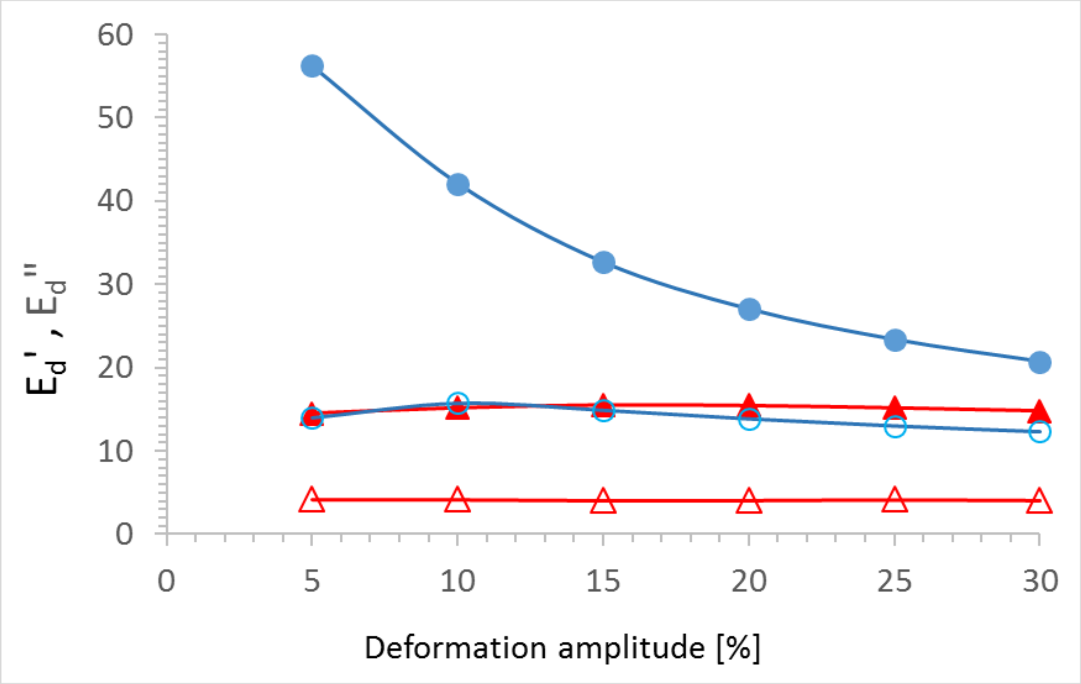

Figure S3. Dilatational storage (closed symbols) and loss modulus (open symbols) for WPI (spheres) and PPI (triangles) stabilized a/w interfaces.

Complete list of fit parameters for step expansion experiments for nanoparticles

In Tables S1 we present the complete list of fit parameters for the nanoparticles in step expansion and compression. For completeness we repeat equation (1) here:

$$\gamma(t) = ae^{-(t/\tau_1)^{\beta}} + be^{-t/\tau_2} + c$$

Table S1. Model parameters a, b, c, τ_1, τ_2, and β for all particles in step expansion-compression.

Nanotubes							
C (mg/ml)	a	b	c	τ_1	τ_2	β	R^2
Expansion							
0.01	1.079	3.315	37.17	186.74	8271.30	0.46	0.9226
0.025	1.444	1.9	32.24	59.10	923.36	0.54	0.9886
0.05	2.415	2.474	20.77	99.80	1014.51	0.46	0.9921
0.1	4.137	0.2127	19.01	62.5	161.86	0.46	0.9979
0.5	3.383	1.314	17.37	17.19	3077.87	0.68	0.9893
1	3.396	0.339	17.65	9.41	398.25	0.72	0.9730
Compression							
0.01	-0.849	1.785	37.15	39.90	37864	0.48	0.5355
0.025	-1.177	-0.325	28.76	16.46	167.73	0.51	0.8836
0.05	-2.491	-1.063	19.9	41.68	355.87	0.65	0.9914
0.1	-1.539	-1.976	18.5	43.57	191.72	1.00	0.9962
0.5	-3.287	-1.886	20.06	29.04	39557	0.85	0.9863
1	-2.548	-0.7065	17.41	15.84	59.77	1.00	0.9668
Cross-linked nanotubes							
C (mg/ml)	a	b	c	τ_1	τ_2	β	R^2
Expansion							
0.025	1.105	1.669	32.92	34.09	739.64	0.59	0.9749
0.05	1.621	1.506	32.22	30.84	677.97	0.53	0.9709
0.1	2.398	1.061	25.56	25.73	1410.44	0.53	0.9822
0.5	2.129	1.319	18.84	15.66	484.73	0.54	0.9422
1	2.213	0.877	17.27	12.08	640.20	0.55	0.9796
Compression							
0.025	-0.701	0.007	33.33	169.23	8481.8	1.00	0.704
0.05	-0.803	-0.479	30.82	19.84	191.50	1.00	0.893
0.1	-2.680	-0.688	23.5	19.26	294.20	0.55	0.993
0.5	-1.490	-1.513	17.59	67.98	306.75	1.00	0.982
1	-1.717	-1.166	16.52	31.15	140.51	0.95	0.995

Table S1 (continued)

Small nanospheres							
C (mg/ml)	a	b	c	τ_1	τ_2	β	R^2
Expansion							
0.01	1.02	1.886	36.50	32.04	832.64	0.4942	0.9712
0.025	2.127	2.591	19.51	16.58	339.56	0.6923	0.9952
0.05	4.168	1.049	18.5	45.25	450.45	0.5396	0.9967
0.1	4.637	0.6584	19.07	46.60	810.37	0.5132	0.9940
0.5	4.191	0.5273	17.81	19.52	437.25	0.5545	0.9973
1	3.888	0.525	17.36	11.57	373.00	0.5963	0.9865
Compression							
0.01	-0.484	-2.339	35.94	101.66	11396	1.00	0.6376
0.025	-1.409	-1.782	20.08	59.14	493.58	0.87	0.9897
0.05	-0.975	-2.177	18.79	45.91	321.85	1.00	0.9838
0.1	-0.989	-2.380	18.85	57.11	195.39	1.00	0.9768
0.5	-1.062	-2.392	17.38	62.89	173.73	1.00	0.9968
1	-2.883	-0.546	17.25	47.87	155.96	1.00	0.9965
Cross-linked small nanospheres							
C (mg/ml)	a	b	c	τ_1	τ_2	β	R^2
Expansion							
0.01	1.038	1.434	36.21	42.39	755.86	0.5128	0.9397
0.025	2.7	1.421	28.00	115.38	821.02	0.6794	0.9978
0.1	2.689	2.2	18.92	19.03	380.23	0.5716	0.9958
0.5	3.21	0.5856	19.33	19.20	529.66	0.6362	0.9908
1	2.753	0.420	18.00	12.14	575.37	0.5788	0.9752
Compression							
0.01	-0.558	-3.025	38.11	73.10	65488	0.89	0.400
0.025	-0.737	-0.660	24.07	86.96	453.31	1.00	0.833
0.05	-1.147	-1.916	21.36	57.74	379.65	1.00	0.977
0.1	-1.287	-2.272	21.16	17.08	185.32	1.00	0.991
0.5	-1.498	-1.730	19.01	11.97	51.57	1.00	0.991
1	-2.323	-0.720	17.82	21.48	105.12	1.00	0.952

Table S1 (continued)

Big nanospheres							
C (mg/ml)	*a*	*b*	*c*	τ_1	τ_2	β	R^2
Expansion							
0.01	0.9044	2.00	36.75	60.20	1082.95	0.5844	0.9869
0.025	2.053	3.388	20.47	66.76	738.01	0.6032	0.9960
0.05	3.548	0.8121	18.70	38.15	348.80	0.5884	0.9968
0.1	4.101	0.4075	17.67	25.28	499.75	0.5811	0.9934
0.5	3.267	0.6043	16.85	10.66	333.56	0.6628	0.9960
1	2.819	0.360	16.49	6.82	167.06	0.6744	0.9875
Compression							
0.01	-0.523	-0.355	34.09	110.31	2886.0	1.00	0.6487
0.025	-1.809	-1.461	19.76	60.35	449.64	0.85	0.99
0.05	-1.961	-1.521	18.41	84.03	250.94	1.00	0.9809
0.1	-1.074	-2.045	17.47	35.21	89.05	1.00	0.9883
0.5	-3.012	-0.188	16.74	34.05	500.0	1.00	0.9867
1	-2.920	-0.071	16.38	23.57	1000.0	1.00	0.9632
Cross-linked big nanospheres							
C (mg/ml)	*a*	*b*	*c*	τ_1	τ_2	β	R^2
Expansion							
0.025	1.231	2.932	25.58	29.43	1031.14	0.7978	0.9780
0.05	1.52	3.148	21.94	41.08	481.00	0.6802	0.9926
0.1	2.424	2.594	18.68	25.48	466.85	0.6032	0.9978
0.5	3.673	1.104	19.33	14.78	862.81	0.5826	0.9791
1	2.976	0.721	18.13	9.80	509.42	0.6986	0.9843
Compression							
0.01	-0.325	-0.434	29.98	84.96	406.01	1.00	0.750
0.025	-1.494	-1.338	20.95	18.83	467.51	0.58	0.974
0.05	-1.364	-1.902	20.49	44.88	365.76	1.00	0.965
0.1	-1.315	-2.279	20.68	20.11	171.06	1.00	0.985
0.5	-2.487	-1.007	19.16	17.05	89.13	1.00	0.983
1	-2.291	-0.803	17.87	13.23	66.23	1.00	0.980

Surface tension as a function of bulk concentration for nanoparticles

In Figure S4 we have plotted the surface stress, measured just before the step expansion, as a function of bulk concentration of nanoparticles, for all 6 particle types. The plotted surface stress is an average of the last 30 data points prior to the step change in surface area (10 or 20%). Error margins are of the order of the symbol size and are not shown. The dashed curve is a schematic plot of a typical surface stress-bulk concentration isotherm, where the plateau value at low concentrations was set equal to the measured surface stress of the bare hexadecane-water interface (51.2 ± 0.2 mN/m). We see that most of our chosen concentrations are in the concentrated regime, where the variations of surface stress with bulk concentration are small. The weak dependence of surface stress on bulk concentration explains why we observe only small variations in β and τ_1 with increasing bulk concentration and cannot observe the onset of the dynamic heterogeneity. Accurate determination of the stretched exponent and τ_1 in the dilute regime was not possible due to increased noise in the surface stress signal.

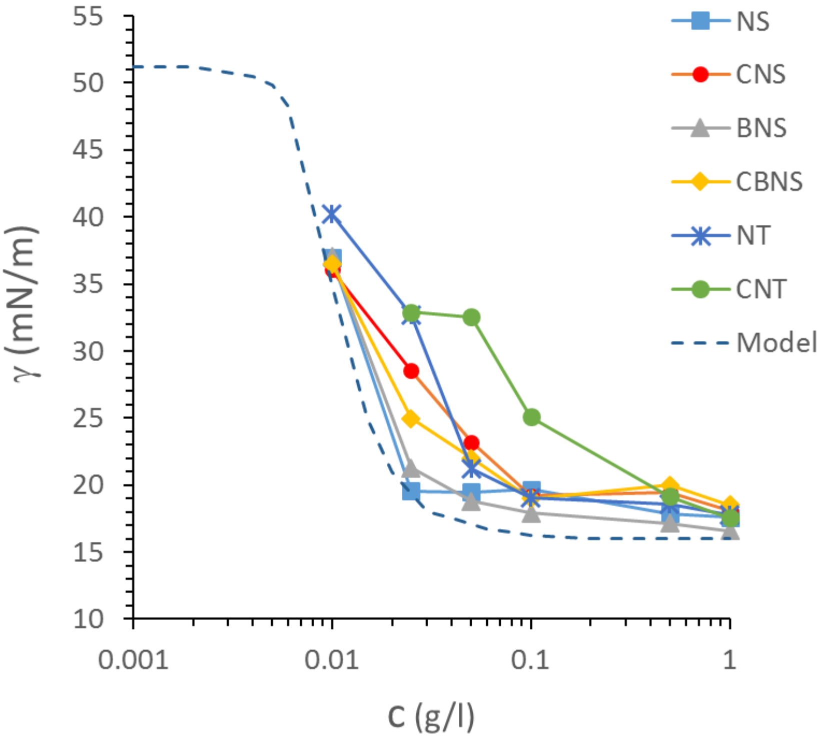

Figure S4. Surface stress as a function of bulk concentration of nanoparticles. The dashed curve is a schematic representation of a complete isotherm, where the plateau value at low concentrations was set equal to the measured equilibrium surface tension of the bare hexadecane-water interface.

Molecular Dynamics Simulations

Equilibrium simulations

In this section we present some simulation results for different microstructures, formed by model block-copolymers at the liquid-vapor interface. The expressions for the interaction potentials are given in equations (2) through (5) in the methods section of the main text, and the parameters in these equations are summarized in tables S2 and S3.

Table S2. Non-bonded interaction potential parameters and cut-off distances used in molecular dynamics simulations.

Structure	Linear strands (Triblock)		In-plane clusters (Diblock)		3D clusters (Diblock)		3D film (Diblock)	
Parameters / Pairs	$\varepsilon_{ij} / \varepsilon$	$r_{\mathrm{cut},ij} / \sigma$	$\varepsilon_{ij} / \varepsilon$	$r_{\mathrm{cut},ij} / \sigma$	$\varepsilon_{ij} / \varepsilon$	$r_{\mathrm{cut},ij} / \sigma$	$\varepsilon_{ij} / \varepsilon$	$r_{\mathrm{cut},ij} / \sigma$
H-H	0.8	2.5	0.2	2.5	0.2	2.5	0.2	2.5
H-T	1.0	$2^{1/6}$	1.0	$2^{1/6}$	1.0	$2^{1/6}$	1.0	$2^{1/6}$
H-W	0.8	2.5	0.5	2.5	0.8	2.5	0.8	2.5
T-T	0.2	2.5	0.8	2.5	0.8	2.5	0.2	2.5
T-W	0.2	2.5	1.0	$2^{1/6}$	1.0	$2^{1/6}$	1.0	$2^{1/6}$
W-W	1.0	2.5	1.0	2.5	1.0	2.5	1.0	2.5

Table S3. Bond (u_b), angle (u_a) and dihedral (u_d) interaction potential parameters used in molecular dynamics simulations.

Interaction potential	u_b	u_a	u_d
Parameters	$k_b = 100\ \varepsilon/\sigma^2$	$k_a = 100\ \varepsilon/\mathrm{rad}^2$	$k_d = 50\ \varepsilon$
	$l_0 = 1.0\ \sigma$	$\theta_0 = \pi/3$ rad	$d = +1$

Apart from calculating thermodynamic properties such as the temperature and the potential energy as a function of simulation time to check for thermal equilibrium, we have calculated local pressure tensor components[5] to check for mechanical equilibrium of the system. The condition of mechanical equilibrium in interfacial systems restricts the pressure tensor to be diagonal and only a function of z (the direction normal to the interface plane), with a constant normal component $P_N(z)$ (i.e. the vapor pressure) throughout the system, and a transverse component $P_T(z)$ which is equal to the vapor pressure in the liquid and vapor bulk phases, but different from that value in the interfacial regions (*1*). The pressure tensor components calculated in our simulations and shown in Figure S5, are in agreement with this within the numerical accuracy.

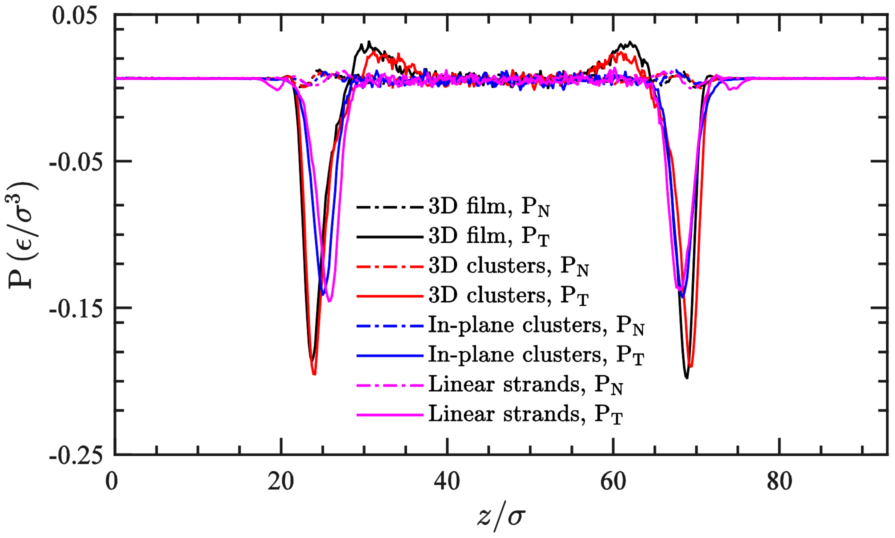

Figure S5 Normal $P_N(z)$ and transverse $P_T(z)$ components of the pressure tensor throughout the simulation box, for different observed microstructures.

Figure S6 shows the monomer number density profiles for the $H_{30}T_{10}$ diblock-copolymers (for 3D film, 3D clusters and in-plane clusters with an average polymer surface concentration of $\Gamma = 150/(2L_xL_y) = 0.029\sigma^{-2}$) and the $T_5H_{10}T_5$ triblock-copolymers (for linear strands with an average polymer surface concentration of $\Gamma = 400/(2L_xL_y) = 0.078\sigma^{-2}$) at the liquid-vapor interface. The inset shows the solvent density profiles for different microstructures where $\rho_l \approx 0.77\sigma^{-3}$ and $\rho_v \approx 0.01\sigma^{-3}$. As can be seen in this figure, for 3D films and 3D clusters, the chains protrude more into the liquid bulk phase than for the linear strands and in-plane clusters. (See also the top row of Figure 4, where the side view of the interfaces is shown for different microstructures.)

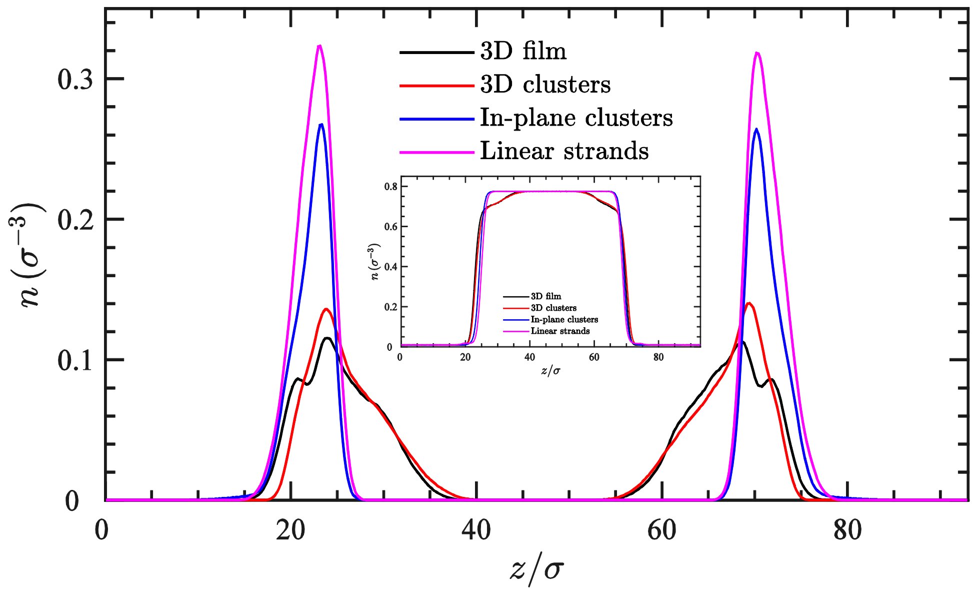

Figure S6 Monomer density profiles for different observed microstructures. The inset shows liquid-vapor density profiles throughout the simulation box for different cases.

In order to evaluate and compare the interfacial in-plane microstructures (see also the bottom row in Figure 4 (main text), where the top view of the interface is shown for different microstructures.) we have calculated the in-plane T block-T block (red beads in Figure 4) center of mass pair correlation functions for $H_{30}T_{10}$ diblock-copolymers in Figure S7. In the case of 3D and in-plane clusters, the pair correlation function profiles have two peaks which correspond to the cluster size and cluster spacing. The pair correlation function for the 3D film is nearly flat, suggesting that there is no lateral ordering in the interface plane for this case.

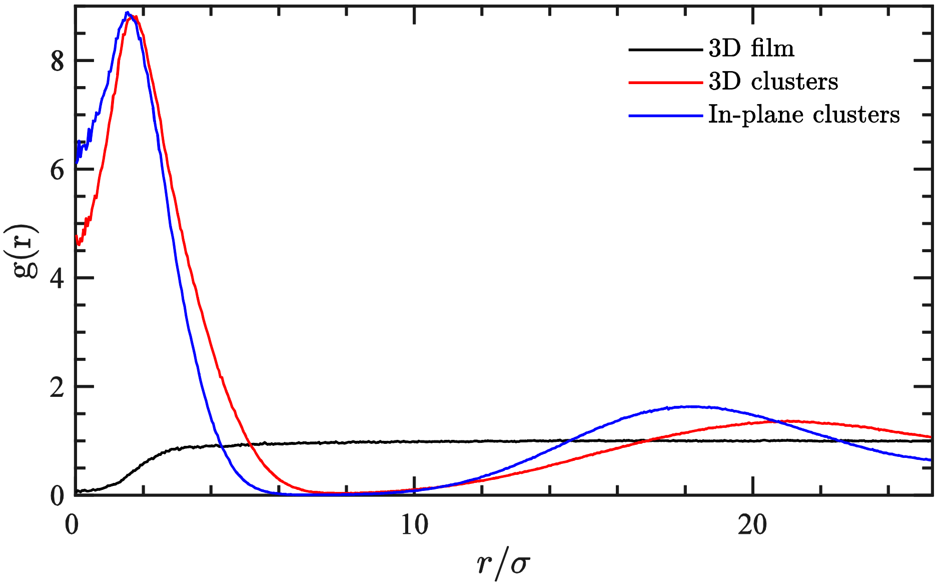

Figure S7. In-plane T block-T block center of mass pair correlation function for $H_{30}T_{10}$ diblock-copolymers at an average surface concentration of $\Gamma = 0.029\sigma^{-2}$.

Nonequilibrium simulations

Our NEMD simulations are carried out by using the SLLOD[6] equations of motion with Lees-Edwards[7] boundary conditions in an NVT ensemble. The velocity gradient tensor used in the SLLOD equations of motion is given by:

$$\nabla v^T = \begin{pmatrix} 0 & 0 & \dot{\gamma} \\ 0 & 0 & 0 \\ 0 & 0 & 0 \end{pmatrix} \quad \text{(S.17)}$$

Here $\dot{\gamma}$ is the imposed shear rate. We have performed NEMD simulations for systems consisting of symmetric diblock-copolymers with different lengths (H_5T_5, $H_{10}T_{10}$ and $H_{15}T_{15}$) at the same surface concentration of $\Gamma = 800/(2L_xL_y) = 0.156\sigma^{-2}$. Figure 5 (main text) shows a snapshot of the triclinic simulation box under shear deformation together with velocity field and the density profile in the direction normal to the interface (z-direction). It can be easily seen that velocity increases monotonically with a constant gradient in the bulk phases while it retards near the interfacial region such that in this region the velocity gradient is almost zero.

In order to observe the symmetry in the system, we consider Figure S8, where density profiles of all species in the system are shown. We simply select the Gibbs dividing surface to be the surface in the

interfacial region with respect to which the densities are symmetric. For the systems under investigation, the Gibbs dividing surface is located at $z_{\mathrm{GDS}} \approx \frac{L_z}{4} \approx 23.2\sigma$.

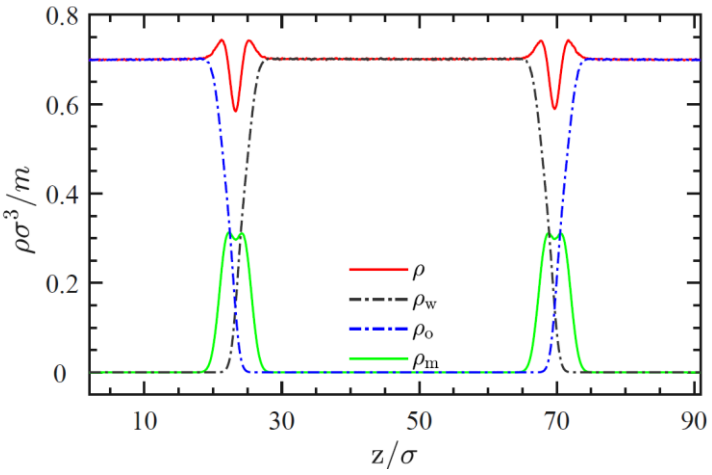

Figure S8. Density profiles of all species for the system consisting of H_5T_5 diblock-copolymers under an imposed shear rate of $\dot{\gamma}_{\mathrm{imp}} = 0.02$ (in LJ reduced units). ρ and ρ_{m} show the particle density (including W, O, H and T) and monomer number density (H and T segments) respectively.

In order to obtain the effective friction coefficient ζ_{xx} from Equation S.16, we have calculated the local shear stress $\sigma_{xz}(z)$ from the volume averaging method (which is an equivalent to the method of planes), as is depicted in Figure S9. It can be easily seen that the shear stress is uniform (within the statistical fluctuations) throughout the simulation box, consistent with the steady state condition.

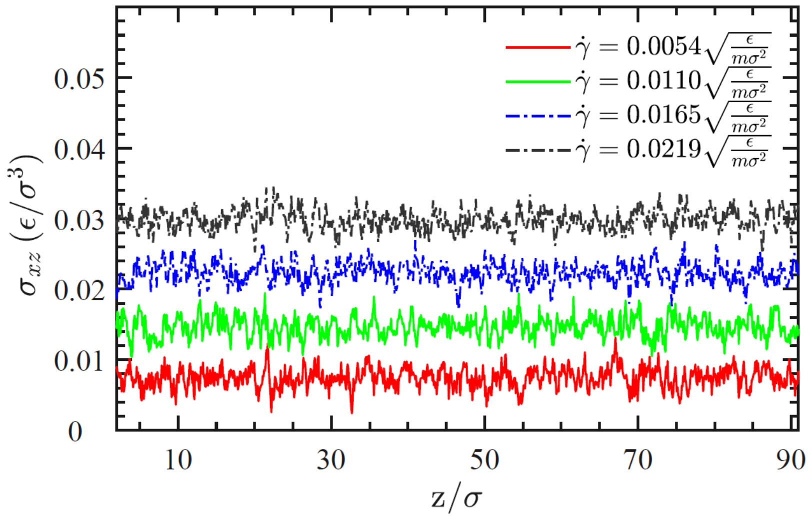

Figure S9. Shear stress profiles for the system consisting of H_5T_5 diblock-copolymers at different shear rates.

References

1. Sagis, L. M. C. Dynamic properties of interfaces in soft matter: Experiments and theory. *Rev. Mod. Phys.* **83**, 1367-1403 (2011).

2. Lamorgese, A., Mauri, R. & Sagis, L. M. C. Modeling soft interface dominated systems: A comparison of phase field and Gibbs dividing surface models. *Phys. Rep*. **675**, 1–54 (2017).
3. J. C. Slattery, L. M. C. Sagis, E. S. Oh, *Interfacial Transport Phenomena* (Springer-Verlag, New York, ed. 2, 2007).
4. L. M. C. Sagis, H. C. Öttinger, Dynamics of multiphase systems with complex microstructure. I. development of the governing equations through nonequilibrium thermodynamics. *Phys. Rev. E* **88**, 022149 (2013).
5. D. J. Evans, G. P. Morris, Nonlinear-response theory for steady planar Couette flow. *Phys. Rev. A* **30**, 1528 (1984).
6. A. W. Lees, S. F. Edwards, The computer study of transport processes under extreme conditions. *J. Phys. C* **5**, 1921 (1972).
7. D. M. Heyes, E. R. Smith, D. Dini, T. A. Zaki, The equivalence between volume averaging and method of planes definitions of the pressure tensor. *J. Chem. Phys.* **135**, 024512 (2011).
